# Prone positioning may increase lung overdistension in COVID-19-induced ARDS

**DOI:** 10.1038/s41598-022-20881-6

**Published:** 2022-10-03

**Authors:** Michal Otáhal, Mikuláš Mlček, João Batista Borges, Glasiele Cristina Alcala, Dominik Hladík, Eduard Kuriščák, Leoš Tejkl, Marcelo Amato, Otomar Kittnar

**Affiliations:** 1 Department of Anaesthesiology, Resuscitation and Intensive Medicine, First Faculty of Medicine, General University Hospital in Prague, Charles University, Prague, Czech Republic; 2 Institute of Physiology, First Faculty of Medicine, Charles University, Albertov 5, 128 00 Prague, Czech Republic; 3 Pulmonology Division, Cardiopulmonary Department, Heart Institute, University of Sao Paulo, São Paulo, Brazil

**Keywords:** Medical research, Physiology, Respiration

## Abstract

Real-time effects of changing body position and positive end-expiratory pressure (PEEP) on regional lung overdistension and collapse in individual patients remain largely unknown and not timely monitored. The aim of this study was to individualize PEEP in supine and prone body positions seeking to reduce lung collapse and overdistension in mechanically ventilated patients with coronavirus disease (COVID-19)-induced acute respiratory distress syndrome (ARDS). We hypothesized that prone positioning with bedside titrated PEEP would provide attenuation of both overdistension and collapse. In this prospective observational study, patients with COVID-19-induced ARDS under mechanical ventilation were included. We used electrical impedance tomography (EIT) with decremental PEEP titration algorithm (PEEP_EIT-titration_), which provides information on regional lung overdistension and collapse, along with global respiratory system compliance, to individualize PEEP and body position. PEEP_EIT-titration_ in supine position followed by PEEP_EIT-titration_ in prone position were performed. Immediately before each PEEP_EIT-titration_, the same lung recruitment maneuver was performed: 2 min of PEEP 24 cmH_2_O and driving pressure of 15 cmH_2_O. Forty-two PEEP_EIT-titration_ were performed in ten patients (21 pairs supine and prone positions). We have found larger % of overdistension along the PEEP titration in prone than supine position (*P* = 0.042). A larger % of collapse along the PEEP titration was found in supine than prone position (*P* = 0.037). A smaller respiratory system compliance was found in prone than supine position (*P* < 0.0005). In patients with COVID-19-induced ARDS, prone body position, when compared with supine body position, decreased lung collapse at low PEEP levels, but increased lung overdistension at PEEP levels greater than 10 cm H_2_O.

Trial registration number: NCT04460859.

## Introduction

A majority of critically ill patients with coronavirus disease (COVID-19) develops acute respiratory distress syndrome (ARDS), needs mechanical ventilation for prolonged time, and exhibits high mortality^1,2^. Prone positioning has been indicated in invasively ventilated patients with ARDS and COVID-19^3^. In a recent evidence-based update of the Surviving Sepsis Campaign COVID-19 guidelines^4^, prone ventilation over no prone ventilation is suggested as a weak recommendation for mechanically ventilated adults with COVID-19 and moderate-to-severe ARDS. On the management of COVID-19-induced ARDS, differently of the pre–COVID-19 era, prone position has been widely adopted, even before intubation in patients breathing spontaneously^5,6^. For instance, seventy-six percent of COVID-19-induced ARDS patients from a cohort in Spain were proned (63% with mild ARDS)^7^.

Beyond standard lung-protective ventilation regimes, intermittent prone positioning is suggested to improve gas exchange by reducing the ventilation and perfusion mismatching, but this has not been fully verified in clinical studies in this new disease entity^8^. Another piece of the COVID-19 puzzle^8^ is the impact of delayed application of prone position^1^. In a cohort of 633 COVID-19 adult patients undergoing invasive mechanical ventilation^1^, overall mortality was highest with non-resolution of hypoxemia and lack of oxygenation response to proning. In another cohort with 3988 critically ill patients with COVID-19^2^, positive end-expiratory pressure (PEEP) levels were higher than those reported for the management of moderate-to-severe ARDS in the pre-COVID-19 era and were an independent factor associated with high mortality.

Real-time effects of changing body position and PEEP on regional lung overdistension and collapse in individual patients remain largely unknown and not timely monitored. The aim of this study was to individualize PEEP in supine and prone body positions seeking to reduce lung collapse and overdistension. We hypothesized that prone positioning with bedside titrated PEEP would provide attenuation of both overdistension and collapse in mechanically ventilated patients with COVID-19-induced ARDS.

## Methods

Consecutive patients with COVID-19-induced ARDS in the first days of mechanical ventilation were included. ARDS was defined according to the Berlin definition^9^. COVID-19 was confirmed by positive nasopharyngeal polymerase chain reaction for SARS-CoV-2. Patients were excluded in case of a contraindication to electrical impedance tomography (EIT): pacemaker, implantable defibrillator, skin lesion.

The design was a prospective observational study. The settings were the ICU of the Department of Anaesthesiology, Resuscitation and Intensive Medicine, First Faculty of Medicine, Charles University, General University Hospital in Prague, Czech Republic; and the ICU of the Pulmonology Division, Cardiopulmonary Department, Heart Institute, University of São Paulo, São Paulo, Brazil.

The study was approved by the Ethics Committee of the General University Hospital, Prague (833/20 S-IV), and by the Ethics Committee of the Heart Institute, University of São Paulo, São Paulo (CAAE: 30938720.8.0000.0068). Written informed consent was waived by both ethics committees (see committee names above) owing to the observational nature of the study. We confirm that all the experiment protocol for involving humans was in accordance to guidelines of national/international/institutional or Declaration of Helsinki.

We used EIT with decremental PEEP titration algorithm (PEEP_EIT-titration_)^10^, which provides information on regional overdistension and collapse^11^, along with global respiratory system compliance, to individualize PEEP and body position aiming to minimize ventilator-induced lung injury (VILI) mechanisms, namely collapse and overdistension. EIT is a noninvasive, radiation-free, real-time imaging method that measures regional changes in lung volumes. Lung collapse and overdistension percentages were determined by comparing each EIT pixel-compliance during PEEP_EIT-titration_^11^. Each pixel-compliance was determined by dividing tidal impedance change by the variation in pressure during the respiratory cycle. Thereafter, overdistension was identified when, for a given pixel, aeration increased and compliance worsened. On the other hand, reversal of collapse was identified if aeration increased and compliance improved.

PEEP_EIT-titration_ in supine position followed by PEEP_EIT-titration_ in prone position were performed. The same lung fields were imaged in supine and prone body positions. During all the procedures, the patients were deeply sedated and under muscle paralysis.

Immediately before each PEEP_EIT-titration_, the same lung recruitment maneuver was performed—2 min of PEEP 24 cmH_2_O and driving pressure of 15 cmH_2_O—removing potential carry-over effects. The PEEP_EIT-titration_, which started at a PEEP level of 24 cmH_2_O, were performed with decremental PEEP steps of 2 cmH_2_O each 30 s until reaching a lower PEEP level set by the clinician.

The EIT data of all PEEP_EIT-titration_ were analyzed to quantify the amounts of lung collapse and overdistension, and respiratory system compliance, at each PEEP step.

### Statistical analysis

The Shapiro–Wilk test was used to test data for normality. The two-way repeated measures ANOVA was used to determine if there was a statistically significant interaction effect between our two within-subjects factors on our continuous dependent variable (i.e., whether a two-way interaction exists). Our continuous dependent variable was % overdistension or % collapse or respiratory system compliance. Our two independent variables were position (supine, prone) and PEEP [two within-subjects factors]. Simple and main effects were also tested where appropriated. The Bonferroni adjustment for multiple tests was applied for post hoc comparisons. The statistical analyses were conducted with SPSS (version 25; IBM Corp, IBM SPSS Statistics for Windows, Armonk, NY). Individual *P* values to indicate statistical tests’ significance are reported were relevant.

### Ethics approval and consent to participate

The study was approved by the Ethics Committee of the General University Hospital, Prague (Etická Komise Všeobecné Fakultní Nemocnice v Praze: 833/20 S-IV), and by the Ethics Committee of the Heart Institute, University of Sao Paulo, Sao Paulo (CAAE: 30938720.8.0000.0068). Written informed consent was waived by both ethics committees (see committee names above) owing to the observational nature of the study. We confirm that all the experiment protocol for involving humans was in accordance to guidelines of national/international/institutional or Declaration of Helsinki.

## Results

Forty-two PEEP_EIT-titration_ were performed in ten patients (21 pairs): supine followed by prone position. One patient received a paired titration five times, one patient four times, one patient three times, two patients two times, and the remaining five patients received a paired titration one time. Table 1 shows the patients’ characteristics.

**Table 1:** Patients’ characteristics.

Sex	Age (years)	BMI (kg/m^2^)	DP (cm H_2_O)^a^	Respiratory system compliance (ml/cm H_2_O)^b^	Duration of mechanical ventilation at recruitment (days)
M	44	30.0	14.0	26	3
F	70	24.2	11.8	26	3
F	79	35.3	10.7	26	1
F	67	33.2	7.3	36	1
M	57	31.4	6.5	60	9
M	73	33.9	6.6	54	2
M	52	35.5	7.8	48	10
M	53	34.7	5.7	65	4
M	66	27.1	9.8	43	0
M	79	30.8	8.2	41	0
Mean	64.0	31.6	8.8	42.5	3.3
SD	12.0	3.7	2.7	14.2	3.5

*BMI*  body mass index, *DP* driving pressure.

^a^At PEEP of 12 cm H_2_O in supine position.

^b^At PEEP of 12 cm H_2_O in supine position.

### Overdistension

**Figure 1:**
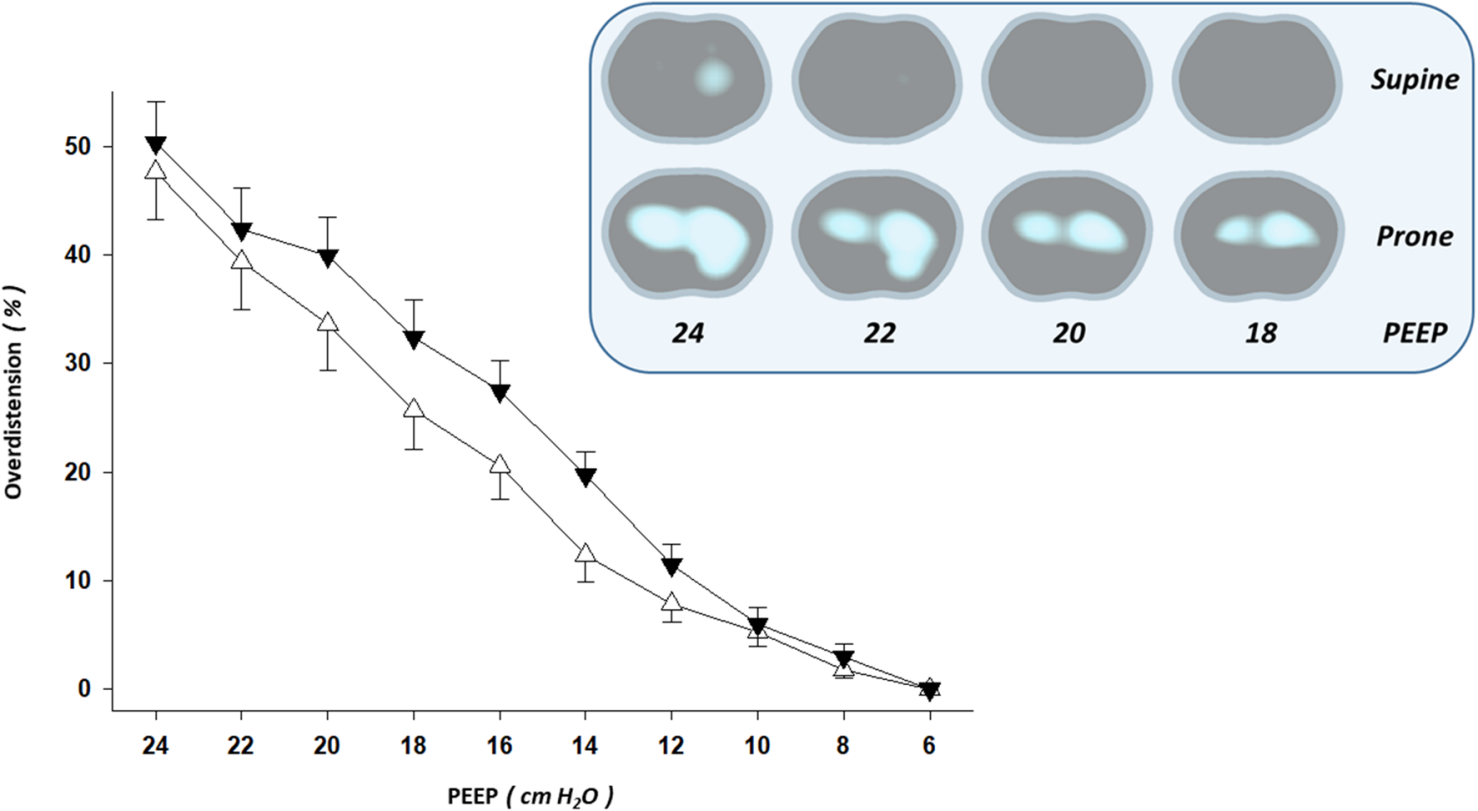
Lung overdistension by electrical impedance tomography in supine vs. prone body position. Line graphs of the electrical impedance tomography (EIT)-based estimations of lung overdistension from forty-two decremental positive end-expiratory pressure (PEEP) titrations—supine vs. prone body position—are shown (mean ± SEM). Some illustrative EIT images of overdistension from one mechanically ventilated patient with coronavirus disease (COVID-19)-induced acute respiratory distress syndrome (ARDS) are also shown: overdistended pixels in white. Note that prone body position increased lung overdistension in comparison with the supine one. Triangle up (white): supine body position. Triangle down (black): prone body position. X axis: decremental PEEP levels of the EIT-PEEP titrations. Y axis: percent of overdistended lung units out of the total lung imaged by EIT.

There was a statistically significant two-way interaction between body position (supine vs. prone) and PEEP on % of overdistension (*P* = 0.042; two-way repeated measures ANOVA). This indicates that the effect of supine position on % of overdistension is different to the effect of prone. That is, % of overdistension changed differently over PEEP levels depending on the position (Fig. 1). We have found larger % of overdistension along the PEEP titration in prone than supine position (that occurred in 70% of the patients). Additionally, when the simple main effects for position were tested, the following results were found: PEEP 20 (*P* = 0.056), PEEP 18 (*P* = 0.055), PEEP 16 (*P* = 0.034), and PEEP 14 cmH_2_O (*P* = 0.040).

### Collapse

**Figure 2:**
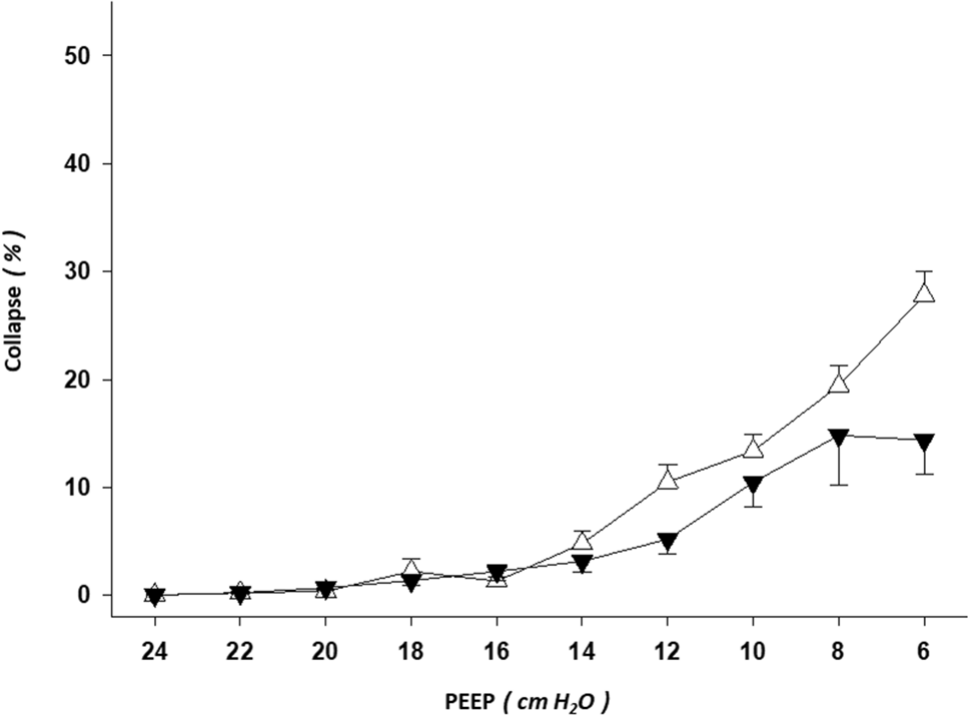
Lung collapse by electrical impedance tomography in supine vs. prone body position. Line graphs of the electrical impedance tomography (EIT)-based estimations of lung collapse from forty-two decremental positive end-expiratory pressure (PEEP) titrations—supine vs. prone body position—are shown (mean ± SEM). Triangle up (white): supine body position. Triangle down (black): prone body position. X axis: decremental PEEP levels of the EIT-PEEP titrations. Y axis: percent of collapsed lung units out of the total lung imaged by EIT.

There was a statistically significant two-way interaction between body position and PEEP on % of collapse (*P* = 0.037; two-way repeated measures ANOVA). This shows that the effect of supine position on % of collapse is different to the effect of prone, i.e. the amount of collapse changed differently over PEEP levels depending on the position (Fig. 2). A larger % of collapse along the PEEP titration was found in supine than prone position. When the simple main effects for position were tested, the following results were found: PEEP 12 (*P* = 0.008), PEEP 10 (*P* = 0.016), and PEEP 6 cmH_2_O (*P* = 0.033).

### Compliance

**Figure 3:**
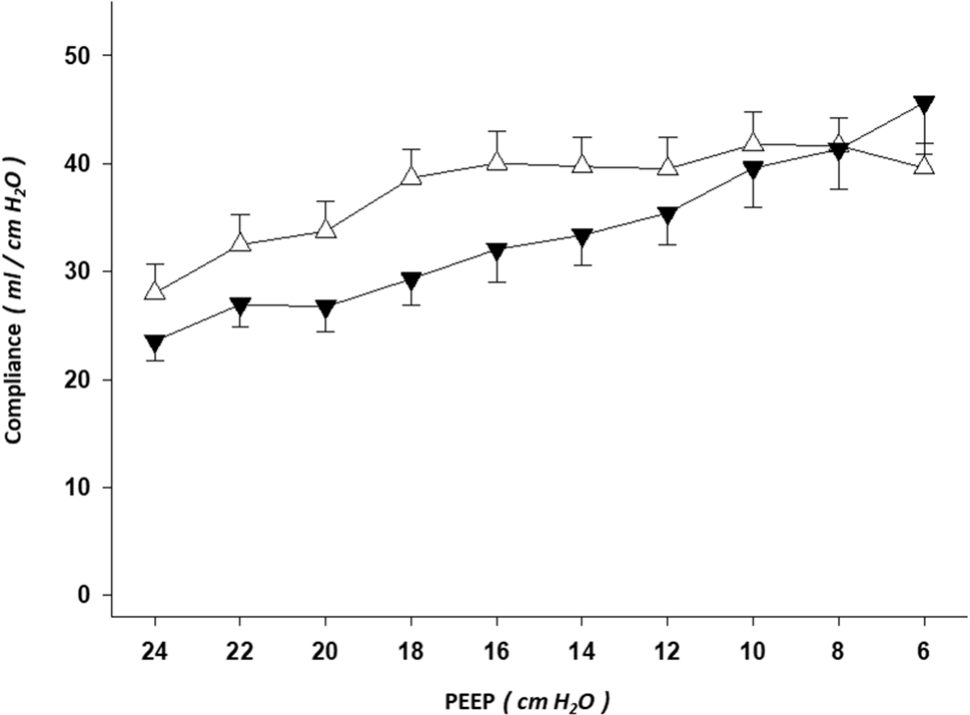
Respiratory system compliance in supine vs. prone body position. Line graphs of the respiratory system compliance from forty-two decremental positive end-expiratory pressure (PEEP) titrations—supine vs. prone body position—are shown (mean ± SEM). Triangle up (white): supine body position. Triangle down (black): prone body position. X axis: decremental PEEP levels of the EIT-PEEP titrations. Y axis: respiratory system compliance.

There was a statistically significant two-way interaction between body position and PEEP on respiratory system compliance (*P* < 0.0005; two-way repeated measures ANOVA). This points out that the effect of supine position on respiratory system compliance is different to the effect of prone. A smaller respiratory system compliance was found in prone than supine position (Fig. 3). Regarding the simple main effects for position, the following results were found: PEEP 20 (*P* < 0.0005), PEEP 18 (*P* < 0.0005), PEEP 16 (*P* < 0.0005), PEEP 14 (*P* = 0.002), PEEP 12 (*P* = 0.032), and PEEP 6 cmH_2_O (*P* = 0.029); in these simple main effects for position analyzes, a smaller respiratory system compliance was found in prone position at the PEEP levels of 20, 18, 16, 14 and 12 cmH_2_O.

### Optimum PEEP

The optimum PEEP of each PEEP_EIT-titration_ was defined by the best compromise between pulmonary overdistension and collapse, i.e. the smallest sum of overdistension and collapse. The optimum PEEP was 13.7 ± 4.5 cmH_2_O in supine position and 10.8 ± 4.3 cmH_2_O in prone.

## Discussion

A major focus of mechanical ventilation for COVID-19-induced ARDS is the avoidance of VILI while facilitating gas exchange via lung-protective ventilation. This is a report of using EIT-based PEEP titration in supine and prone body positions to personalize body positioning and PEEP in adult patients with COVID-19-induced ARDS. During prone position, along with a smaller amount of collapse at low PEEP levels, we found a larger amount of overdistension at PEEP levels greater than 10 cm H_2_O.

The primary indications for implementing prone ventilation in patients with ARDS are the need to improve oxygenation and the potential for prone position to reduce mortality^12^. It is usually assumed that in prone position there is less overdistension in nondependent lung regions and less cyclical airspace opening and closing in dependent regions^12^. Our findings suggest that in patients with COVID-19-induced ARDS these beneficial effects cannot be a priori assumed.

Hypoxemia disproportional to mechanics, lack of oxygenation response to proning, and poorly recruitable lungs with increased recruitability with alternating body position between supine and prone have been reported in COVID-19-induced ARDS^1^. A number of hypotheses have been proposed to explain such baffling hypoxemia, including SARS-CoV-2-specific effects on oxygen receptor chemosensitivity, reduced diffusion capacity, and loss of hypoxic vasoconstrictive mechanisms^8^. Many pathophysiological events may affect either lung perfusion or ventilation, all of which could lead to a ventilation/perfusion mismatch. Our findings suggest that, during prone, one of them can be a worsened ventilation/perfusion matching due to larger regional overdistension within more aerated lung regions and, consequently, more diversion of pulmonary blood flow away from these units, such effect surpassing the diminution of regional collapse within less aerated lung regions.

Our data underpin the importance of timely PEEP titrations tackling the dynamically changing phases of this disease^8^. They put forward the importance of monitoring and quantifying in real-time changes in overdistension and collapse—as well as the relevance of personalized PEEP adjustments—every time body positions are changed^10^. The recommendation of applying nonpersonalized low or high PEEP may lead to insufficient and/or excessive PEEP in terms of protection of VILI^8^.

The response of respiratory system compliance to prone position is variable and complex^13^. Several observational studies reported that respiratory system compliance was unaltered or modestly decreased when turning from supine position to prone position in subjects with ARDS^14–19^. Other studies have reported improved respiratory system compliance upon being placed in prone position^20,21^, or after an extended period of prone position^14^. There is also evidence suggesting that patients with extrapulmonary sources of ARDS may be more likely to exhibit decreased respiratory system compliance when placed in prone position^16^ possibly attributable to an accentuation of the characteristically decreased chest wall compliance found in that condition^22^. Our respiratory system compliance findings may suggest that, in prone position, the increase in lung overdistension predominated over the decrease of lung collapse. But we did not measure the changes in chest wall compliance. Anyway, altogether, our data point out that global respiratory mechanics alone does not provide enough information towards an individualization of body position and PEEP aiming at to reduce regional lung collapse and overdistension.

The main limitations include the small sample size, highly selected cohort, and the lack of repeated blood gases with PEEP titration (due to safety measures) as well as the lack of blood gases at optimal PEEP level. Another limitation is the lack of esophageal pressure measurements during prone position, which may change the chest wall elastance; esophageal pressure measurements would allow the partitioning of the respiratory system in the lung and the chest wall components. In addition, we do not provide data after many hours in prone position. Finally, larger series are needed to confirm the present findings.

## Conclusions

In patients with COVID-19-induced ARDS, prone body position, when compared with supine body position, decreased lung collapse at low PEEP levels, but increased lung overdistension at PEEP levels greater than 10 cm H_2_O.

## Data Availability

The data are with the authors and will be available upon reasonable request. The data is available from the corresponding author: Joao Batista Borges.

## Abbreviations

COVID-19: Coronavirus disease
ARDS: Acute respiratory distress syndrome
ICU: Intensive care unit
PEEP: Positive end-expiratory pressure
F_I_O_2_: Fraction of inspired oxygen
PaO_2_: Arterial oxygen partial pressure
PaO_2_/F_I_O_2_: Partial pressure of arterial oxygen ratio
*P*_L_: Transpulmonary pressure (*P*_L_ = airways pressure − pleural pressure)
EIT: Electrical impedance tomography
PEEP_EITtitration_: PEEP titration algorithm using EIT
VILI: Ventilator-induced lung injury
